# Supplementary material for: The red flags of ulnar neuropathy in leprosy
Source: PLoS One. 2021 Nov 19;16(11):e0259804. doi: 10.1371/journal.pone.0259804 (PMC8604365; doi:10.1371/journal.pone.0259804)
Supplement: Attachment — Submitted filename: Response to reviewers.docx [file pone.0259804.s003.docx]

Rio de Janeiro, august 17, 2021

Dr. Leila Harhaus
Academic Editor
PLOS ONE

Dear Dr Harhaus,

We are re-submitting the Manuscript ID PONE-D-21-15552
“The red flags of ulnar neuropathy in leprosy” with corrections as suggested by the academic editor and reviewer. We appreciated the thoughtful suggestions that greatly contributed to improve the manuscript.

**Academic Editor:**

*1 - We perform the modifications to meet the style requirement according to: https://journals.plos.org/plosone/s/file?id=wjVg/PLOSOne_formatting_sample_main_body.pdf and https://journals.plos.org/plosone/s/file?id=ba62/PLOSOne_formatting_sample_title_authors_affiliations.pdf*

*2 – We include the tables as part of the main manuscript and we removed the individual files.*

*3- We removed any funding-related text from the manuscript. We would like to keep the Funding Statement: The authors received no specific funding for this work.*

**Reviwer:**

**Reviwer: The investigative profile used is grossly inadequate. There is no information provided on the use of ultrasound and MRI neurography findings. Both of these have been studied in leprosy and some conclusions are already available.**

*Response: We are grateful for the possibility of discussing the importance of complementary methods in the diagnostic suspicion of the pure neural form of leprosy, including imaging. However, the gold standard for this diagnosis is the histopathological study, which requires an invasive and definitive procedure, a nerve biopsy. The aim of this study was to show that some neurophysiological findings may rise the suspicion of the diagnosis. This way, we will be able to increase the sensitivity and specificity of the indication of nerve biopsy for these patients. We agree that lately, studies evaluating the role of ultrasonography and the findings of magnetic resonance neurography have been available. However, they either describe specific findings in patients with pure neural leprosy. In some articles, the presence of Doppler was described in neuritis. It is important in patients with general leprosy, but pure neural leprosy patients have a lower incidence of acute neuritis, so US findings are less useful in this group of patients.*

**Reviewer : Patients of lepra reactions perhaps should not be considered in this manuscript as the diagnosis, is more clear during the reaction state and the electrophysiology findings are different in relation to the acutely altered immunity.**

*Response: Excellent comment, but patients with leprosy reactions were used precisely because the diagnosis of neuritis is more evident in this group of patients. Neurophysiological and clinical findings are well defined during reactional episodes. Patients with a pure neural leprosy do not have evident clinical findings of neuritis (pain, thickening and sensory dysfunction, associated or not with motor dysfunction), neurophysiological findings help in this diagnosis.*

**Rewier: Minor comments:**

**Introduction could be shortened**

*Response: According to the suggestion of the referee we short a little one the introduction (p. 3, lines 4,5 and 6).*
